# Extracellular vesicles participate in the pathogenesis of sepsis

**DOI:** 10.3389/fcimb.2022.1018692

**Published:** 2022-12-12

**Authors:** Chang Tian, Ke Wang, Min Zhao, Shan Cong, Xin Di, Ranwei Li

**Affiliations:** ^1^ Department of Respiratory and Critical Care Medicine, The Second Hospital of Jilin University, Changchun, Jilin, China; ^2^ Department of Urinary Surgery, The Second Hospital of Jilin University, Changchun, Jilin, China

**Keywords:** extracellular vesicles, exosomes, microvesicles, sepsis, pathogenesis

## Abstract

Sepsis is one of the leading causes of mortality worldwide and is defined as life-threatening organ dysfunction caused by a dysregulated host response to infection. The early diagnosis and effective treatment of sepsis still face challenges due to its rapid progression, dynamic changes, and strong heterogeneity among different individuals. To develop novel strategies to control sepsis, a better understanding of the complex mechanisms of sepsis is vital. Extracellular vesicles (EVs) are membrane vesicles released from cells through different mechanisms. In the disease state, the number of EVs produced by activated or apoptotic cells and the cargoes they carry were altered. They regulated the function of local or distant host cells in autocrine or paracrine ways. Current studies have found that EVs are involved in the occurrence and development of sepsis through multiple pathways. In this review, we focus on changes in the cargoes of EVs in sepsis, the regulatory roles of EVs derived from host cells and bacteria, and how EVs are involved in multiple pathological processes and organ dysfunction in sepsis. Overall, EVs have great application prospects in sepsis, such as early diagnosis of sepsis, dynamic monitoring of disease, precise therapeutic targets, and prevention of sepsis as a vaccine platform.

## 1 Introduction

According to the latest guidelines, sepsis was defined as a dysregulated host immune response caused by infection, which in turn causes a systemic inflammatory response and even multiple organ dysfunction (Singer et al., 2016). Globally, the incidence of sepsis was 189 hospitalized sepsis cases per 100,000 person-years, and the mortality rate is approximately 26.7%, presenting an enormous economic burden worldwide (Fleischmann-Struzek et al., 2020).

Extracellular vesicles (EVs) are membrane vesicles that can be secreted by all cell types and are a novel signal transduction method discovered recently. Currently, they are considered to be divided into three main categories: exosomes, microvesicles and apoptotic bodies, and this classification is mainly based on their biogenesis process (van Niel et al., 2018). That is, microvesicles were formed by budding and fission directly from the plasma membrane, while exosomes were formed by a unique mechanism, mainly through two invaginations of the plasma membrane, and finally existed in maturation multivesicular bodies (MVB) in the form of intraluminal vesicles (ILVs), which are then released extracellularly by fusion with the plasma membrane (van Niel et al., 2018). Most of the current research still focuses on exosomes, mainly because of the particularity of their biogenesis mechanism, which many scholars believe may be related to unique functions. However, the biological study of extracellular vesicles is still unclear. The separation and purification techniques and identification methods are still questionable. Although many researchers claim to be studying exosomes or microvesicles, we still refer to them collectively as EVs. It should be noted that in earlier studies, the vesicle-like structures present outside the cell were called microparticles (MPs). Therefore, in this paper, MPs from earlier studies were also included in the category of EVs.

In recent years, a large number of original studies on EVs in sepsis have been published. In sepsis, EVs derived from activated or apoptotic cells were altered in both the number and cargoes they carry, regulating signal transduction and altering the phenotype of neighboring cells in an autocrine or paracrine manner (Juan et al., 2021; Murao et al., 2021; Wang et al., 2021). Here, we mainly explore the changes in the cargoes of EVs in sepsis, the regulatory role of EVs from different cell sources, and how EVs are involved in different pathological processes and organ dysfunction in sepsis.

## 2 Cargoes of EVs in sepsis

**Figure 1:**
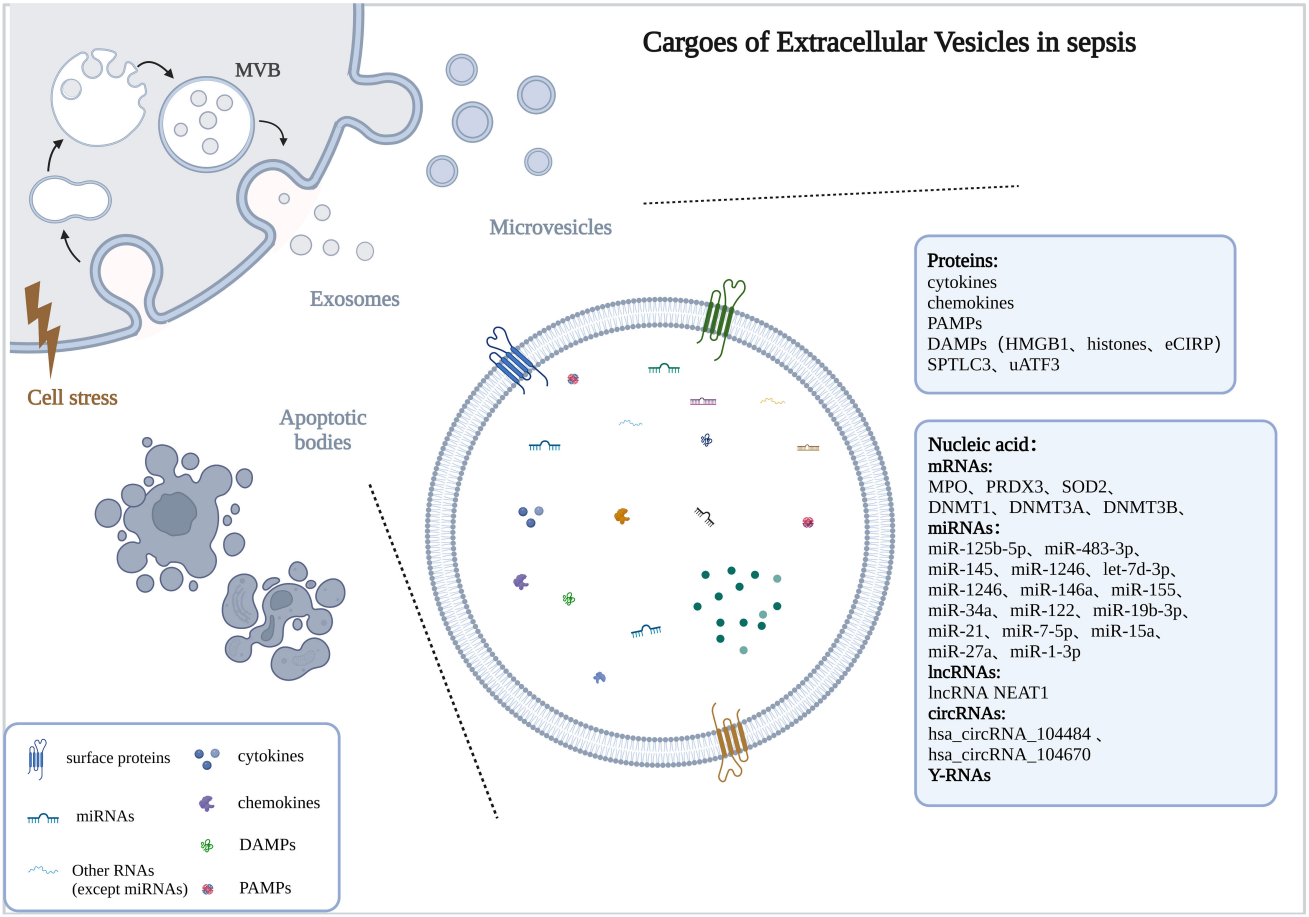
Cargoes of Extracellular Vesicles in sepsis. The drawings were created with BioRender.com. multivesicular bodies (MVBs).

The number of EVs was significantly increased in sepsis or stimulated by bacteria (Dakhlallah et al., 2019; Timár et al., 2013) and was positively correlated with the severity of sepsis (Dakhlallah et al., 2019). Here, we described alterations in the cargoes carried by EVs in sepsis (**Figure 1**).

### 2.1 Proteins

Studies have found that the protein profiles carried by EVs in the body fluids of sepsis were altered and may be associated with disease progression. In the early stages of sepsis, acute phase reactive proteins and immunoglobulins involved in inflammatory responses were upregulated (Morris et al., 2020). With the progression of the disease and the intensification of the inflammatory response, the levels of inflammation-related proteins changed dynamically, among which the protein SPTLC3 involved in sphingolipid metabolism was negatively correlated with body temperature and C-reactive protein (CRP) (Xu et al., 2018). Current studies have found that EVs with altered protein profiles originated from a variety of cell types, including activated macrophages (Wang et al., 2019), monocytes (Homsy et al., 2019; Wisler et al., 2020), neutrophils (Timár et al., 2013), etc.

In the serum of septic mice, a variety of cytokines/chemokines were specifically encapsulated into exosomes, and the use of the exosome inhibitor GW4869 significantly reduced the release of exosomes and pro-inflammatory cytokines (Essandoh et al., 2015). Cytokines/chemokines in exosomes often show delayed peaks (12-24h), unlike the peak time of serum-free cytokines/chemokines (2-12h), and they may be involved in the regulation of lymphocyte differentiation, proliferation, and chemotaxis (Gao et al., 2019). Cytokine levels were also elevated in LPS-stimulated macrophage-derived exosomes (McDonald et al., 2014). Current studies have shown that EVs released from various cell types carried damage associated molecular patterns (DAMPs), such as high mobility group box 1 protein (HMGB1), histones, and extracellular cold-induced RNA-binding protein (eCIRP), which were mainly derived from macrophages (Nair et al., 2018; Murao et al., 2021; Wang et al., 2021; Yang et al., 2022), others include platelets (Jiao et al., 2020) and hepatocytes (Li et al., 2020).

### 2.2 Nucleic acid

EVs are important carriers for nucleic acid transport, which can protect nucleic acids from being degraded by nucleases and maintain their stability. The types and contents of nucleic acids encapsulated by EVs were changed in sepsis, including mRNA, miRNA, lncRNA, circRNA, etc.

The levels of mRNAs related to antioxidant defense and oxidative stress were up-regulated in EVs of sepsis patients (Real et al., 2018). In addition, DNMT1, DNMT3A, and DNMT3B mRNA were also up-regulated in EVs and correlated with the severity and prognosis of sepsis (Dakhlallah et al., 2019).

The miRNA expression profiles of EVs were altered in sepsis (McDonald et al., 2014; Goodwin et al., 2015; Reithmair et al., 2017; Real et al., 2018; Xu et al., 2018), and may be associated with the risk, severity and prognosis of sepsis (Balusu et al., 2016; Real et al., 2018; Hermann et al., 2020; Qiu et al., 2022). These miRNAs are involved in sepsis from multiple pathways, including immune regulation, microvascular dysfunction, and organ dysfunction (Fan et al., 2014; Goodwin et al., 2015; Wang et al., 2015; Song et al., 2017; Xu et al., 2018; Zhou et al., 2018; Zhou et al., 2018; Deng et al., 2019; Jiang et al., 2019; Cao et al., 2019; Pan et al., 2019; Lv et al., 2020; Yao et al., 2021; Sun et al., 2021; Gao et al., 2021; Liu et al., 2022).

In EVs of sepsis, other types of non-coding RNAs were also altered, including lncRNAs (Ma et al., 2021; Sui et al., 2021a; Liang et al., 2022; Wei et al., 2022), circRNAs (Cao et al., 2022), and Y-RNA (Driedonks et al., 2020). Studies have shown that lncRNA NEAT1 carried by EVs in sepsis was associated with the aggravation of sepsis-related encephalopathy (Wei et al., 2022), lncRNA TUG1 was involved in promoting macrophage M2 polarization (Ma et al., 2021), and lncRNA-p21 can inhibit LPS-induced lung cells injury, lncRNA IGF2-AS promoted endothelial progenitor cell pyroptosis (Sui et al., 2021a; Liang et al., 2022). Hsa_circRNA_104484 and hsa_circRNA_104670 were up-regulated in serum exosomes of patients with sepsis, which may serve as diagnostic markers for sepsis (Tian et al., 2021). Mmu_circ_0001295 in exosomes was involved in alleviating septic kidney injury (Cao et al., 2022). The cell-type-specific Y-RNA isoform ratios in plasma EVs were altered in a human endotoxemia model and closely correlated with inflammation-induced changes in circulating neutrophil and monocyte numbers (Driedonks et al., 2020).

## 3 EVs derived from different cell types in sepsis

**Figure 2:**
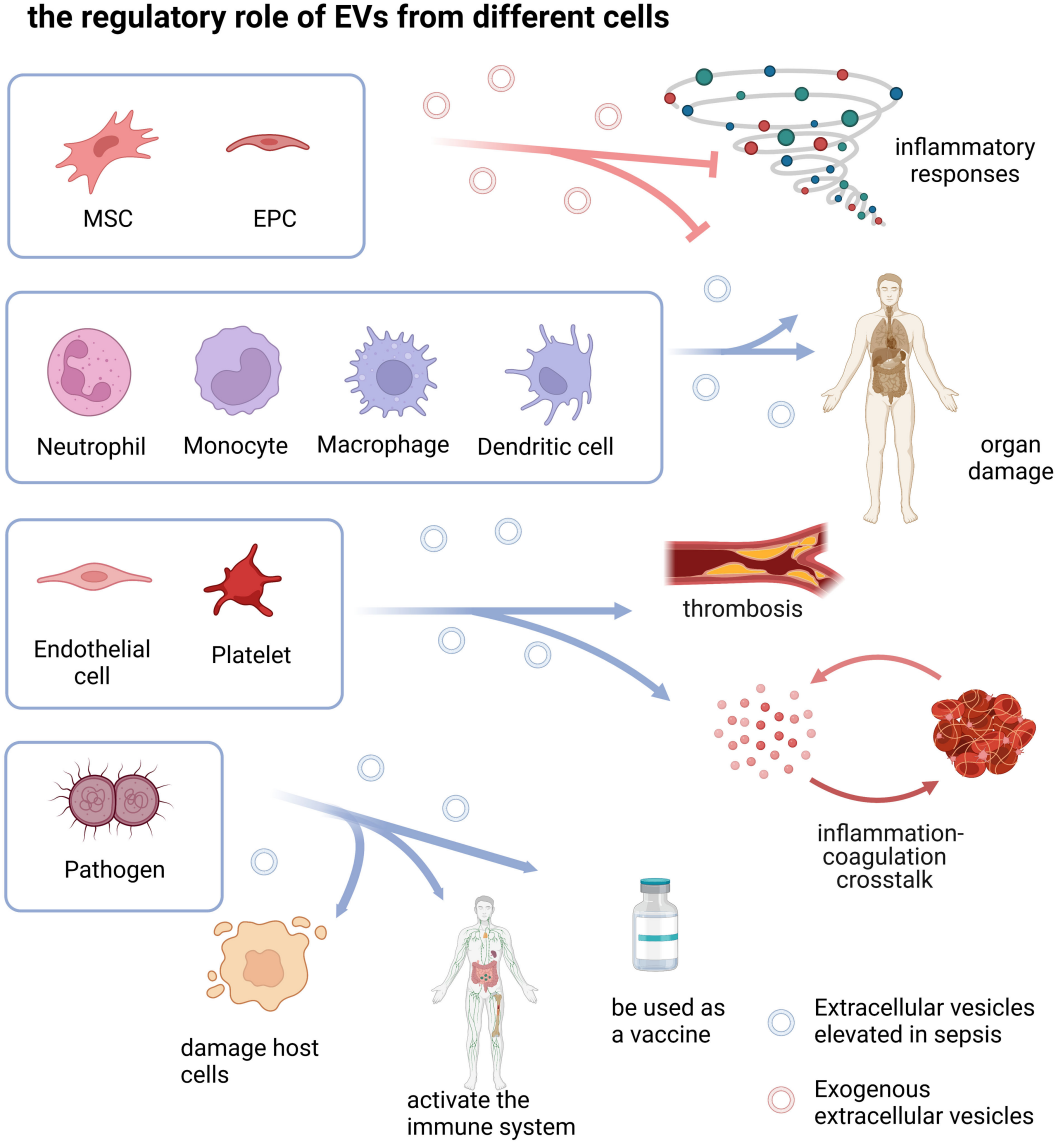
the regulatory role of EVs from different cells. The drawings were created with BioRender.com. Mesenchymal stem cell (MSC), Endothelial progenitor cell (EPC).

EVs are a group of heterogeneous vesicles whose heterogeneity is reflected in size, cargoes, biogenesis, origin, and function (Willms et al., 2018).The cargoes carried were highly correlated with the donor cell, so the function of EVs may be highly correlated with the donor cell (van Niel et al., 2018). Here, we discussed the role of different cell type-derived EVs in sepsis (**Table 1** and **Figure 2**).

**Table 1:** Extracellular vesicles mediated functional crosstalk between distinct cells in sepsis.

ID	Donor cells	Cargoes	Target cells	Signaling pathways/mechanisms	Pathophysiological changes	Reference
1	IL-1β-Primed Mesenchymal Stem Cells	miR-146a	macrophages	ND	Induced macrophage M2 polarization Ameliorated sepsis	(Song et al., 2017)
2	IL-1β-primed-mesenchymal stem cells	miR-21	macrophages	ND	Induced macrophage M2 polarization Ameliorated sepsis	(Yao et al., 2021)
3	BMMSCs	microRNA-27b	macrophages	downregulated JMJD3 inactivated the NF-κB signaling pathway.	Diminished production of pro-inflammatory cytokines	(Sun et al., 2021)
4	BMMSCs	miR-191	macrophages	inhibited the expression of DAPK1	Inhibited the inflammation of alveolar macrophage	(Liu et al., 2022)
5	BMMSCs	miR-223	macrophage cardiomyocytes	reprogramed the protein contents (Sema3A andStat3)of exosomes.	Inhibited the secretion of IL-1β and IL-6 Inhibited cardiomycoyte death Have therapeutic effects on sepsis-induced heart failure and mortality	(Wang et al., 2015)
6	BMMSCs	lncRNA-p21	lung epithelial cells	lncRNA-p21 /miR-181/SIRT1 axis	Suppressed cell apoptosis Alleviate lung tissue injury	(Sui et al., 2021a)
7	BMMSCs	lncRNA IGF2-AS	EPCs	HMGA1/TYMS axis	Reduced the level of dNTP Promoted pyroptosis of EPCs	(Liang et al., 2022)
8	BMMSCs	Ang-1 mRNA	lung microvascular endothelial cell macrophages	ND	Ameliorated the lung inflammation, including the influx of WBCs and neutrophils, and MIP-2 secretion. Suppressed the secretion of TNF-α, and promoted the secretion of IL-10. Beneficial effects on pulmonary capillary permeability	(Tang et al., 2017)
9	BMMSCs	mitochondrial	macrophages	enhanced macrophage oxidative phosphorylation	Inhibited inflammatory cytokine secretion, Increased expression of the M2 marker CD206 Enhanced phagocytic capacity protect against endotoxin-induced lung injury in vivo	(Morrison et al., 2017)
10	BMMSCs	ND	macrophages	inhibited hypoxia-inducible factor 1 (HIF-1)α down-regulated the expression of several essential proteins of glycolysis	Inhibited M1 polarization and promoted M2 polarization Prevented LPS-induced ARDS.	(Deng et al., 2020)
11	BMMSCs	ND	alveolar epithelial cells	Nrf-2/ARE and NF-κB signaling pathways	Reversed LPS-induced ALI	(Li et al., 2020)
12	umbilical cord mesenchymal stem cells	ND	renal tubular epithelial cells	miR-146b/IRAK1/NF-κB axis	Lessened pro-inflammatory response Decreased the serum creatinine (Cr) and blood urea nitrogen (BUN) levels, ameliorated the morphological damage and inhibited renal tubular cells apoptosis. Improved survival in mice with sepsis	(Zhang et al., 2020)
13	ADSCs	ND	macrophages	Notch-miR148a-3p Axis NF-κB pathway	Regulated Polarization of Macrophages Decreased proinflammatory cytokines( IL-1β, IL-6, and TNF-α) Alleviated Sepsis in Mice	(Bai et al., 2020)
14	ADSCs	miR-126	endothelial cells	PI3K/Akt pathway	Inhibited histone-mediated lung hemorrhage and edema Attenuated vascular hyper-permeability in mice.	(Mizuta et al., 2020)
15	EPCs	miRNA-126	small airway epithelial cells	miRNA-126-3p/PIK3R2	Reduced the cell number, protein concentration, and cytokines/chemokines in the bronchoalveolar lavage fluid (BALF) Reduced myeloperoxidase (MPO) activity, lung injury score, and pulmonary edema Protected against lung injury.	(Zhou et al., 2019)
16	EPCs	microRNA-93-5p	proximal tubular cells	KDM6B/H3K27me3/TNF-α axis	Attenuated multiple organ injury, vascular leakage, inflammation, and apoptosis in septic mice.	(He et al., 2020)
17	EPCs	miR-126-3p 、miR-126-5p	HMVECs	ND	Reduced vascular leakage Improved organ function Increased survival	(Zhou et al., 2018)
18	EPCs	lncRNA TUG1	macrophages	TUG1 /miR-9-5p/SIRT1 axis	Induced anti-inflammatory macrophage polarization(promoted M2 macrophage polarization) Suppressed macrophage-medicated inflammatory injury to the pulmonary vascular endothelium	(Ma et al., 2021)
19	neutrophils	active myeloperoxidase	vascular endothelial cells	myeloperoxidase-hydrogen peroxide-chloride system	Induced a loss of cell membrane integrity and morphological changes	(Pitanga et al., 2014)
20	neutrophils	ND	monocytes	ND	Increased activation Increased the phagocytic ability	(Prakash et al., 2012)
21	PMNs	ND	macrophages	ND	down-modulated cellular activation in macrophages. anti-inflammatory effect	(Gasser and Schifferli, 2004)
22	PMNs	ND	macrophages	MerTK pathway	down-modulated the proinflammatory signals	(Eken et al., 2010)
23	PMNs	ND	monocyte-derived dendritic cells	ND	Interfered with maturation Reduced their phagocytic activity Increased the release of TGF-beta1.	(Eken et al., 2008)
24	Mononuclear Phagocytes	caspase-1	HPMVECs	ND	Induced cell apoptosis/death	(Mitra et al., 2015)
25	monocytes	caspase 1 and GSDMD	endothelial cells	ND	Induced cell apoptosis	(Mitra et al., 2018)
26	monocytes	TXNIP-NLRP3	macrophages	ND	promoted the cleavage of inactive IL-1β and IL-18 aggravated cardiovascular inflammation	(Wang et al., 2021)
27	monocytes	mtDAMP	PMNs	ND	Suppressed chemotaxis	(Konecna et al., 2021)
28	monocytes	GLUT-1	HUVECs	ND	Induced inflammatory cytokines	(Yang et al., 2022)
29	macrophages	ND	adrenocortical cells	TREM2	Inhibited corticosterone biosynthesis	(Ye et al., 2021)
30	macrophages	eCIRP	macrophages	ND	Induced inflammation and cytokine production. Promoted Neutrophil Migration chemotaxis effects	(Murao et al., 2021)
31	macrophages	ND	hepatocytes	NLRP3 signaling pathway	liver injury	(Wang et al., 2019)
32	monocytes	ND	monocytes	ND	Reduced TNF-α generation in response to LPS stimulation.	(Wisler et al., 2020)
33	macrophages	ND	macrophages	ND	Induced pro-inflammatory cytokine production(TNF-α、IL-1β、IL-6) Promoted cardiac inflammation and myocardial dysfunction in mice	(Essandoh et al., 2015)
34	macrophages	HMGB1	hepatocytes	NLRP3 inflammasomes signaling pathway	Induced hepatocyte pyroptosis Promoted acute liver injury	(Wang et al., 2021)
35	dendritic cells	MFG-E8	macrophages	ND	Attenuated proinflammatory responses Enhanced phagocytosis	(Miksa et al., 2006)
36	Immature dendritic cells	MFG-E8	macrophages	ND	Enhanced apoptotic cell clearance Attenuated the acute systemic inflammatory response	(Miksa et al., 2009)
37	endothelial cells	c-Src	endothelial cells neutrophils	ND	Increased endothelial cells monolayer permeability to albumin Activated neutrophils and endothelial cells Promoted neutrophil-endothelium adhesion and induced NET production Induced endothelial barrier dysfunction.	(Chatterjee et al., 2020)
38	endothelial cells	ND	endothelial cells	NF-κB pathway	Induced an inflammatory response	(Liu et al., 2017)
39	endothelial cells	HSPA12B	macrophages	NF-κB pathway	Increased IL-10 levels and decreased the production of TNF-α and IL-1β	(Tu et al., 2020)
40	endothelial cells	several miRNAs increased (miR-221-3p, miR-222-3p, miR-221-5p, miR-155-5p, miR-1247-3p, mir-129-5p, miR-148a-5p, and miR-222-5p)	cardiomyocytes	down-regulated the expression of apoptosis-related proteins such as BAK1, P53, and PTEN.	Promoted the survival of cardiomyocytes Enhanced the cell viability and attenuated the injury of cardiomyocytes.	(Cao et al., 2021)
41	choroid plexus epitheliums	ND	brain cells	ND	Acted as a new mechanism of blood-brain communication. Transfered pro-inflammatory message to recipient brain cells.	(Balusu et al., 2016)
42	tubular epithelial cells	miR-19b-3p	macrophages	NF-κB/SOCS-1	Promoted M1 macrophage activation in kidney injury Caused tubulointerstitial inflammation	(Lv et al., 2020)
43	platelets	IL-1β	endothelial cells	ND	Induced VCAM-1 production Promoted endothelial cell activation	(Brown and McIntyre, 2011)
44	platelets	ND	monocytes	CD40/TRAF6/NFκB signalling pathway	Caused aggregate formation Provoked the expression and release of inflammatory mediators, including IL-1β, TNFα, MCP-1 and MMP-9 Enhanced MCP-1-induced monocyte migration	(Bei et al., 2016)
45	platelets	ND	endothelial cells aortic smooth muscle cell lines	NADPH oxidase activity	Induced vascular cell apoptosis	(Janiszewski et al., 2004)
46	platelets	ND	endothelial cells	peroxynitrite generation	Induced cell apoptosis	(Gambim et al., 2007)
47	platelets	HMGB1 and/or miR-15b-5p and miR-378a-3p	PMNs	Akt/mTOR autophagy pathway	Induced NET formation	(Jiao et al., 2020)
48	RBCs	ND	blood leukocytes	ND	Induced a host inflammatory response (Increased the production of TNF, IL-6 and IL-8)	(Straat et al., 2016)
49	erythrocytes	ND	macrophages	TLR4-MyD88-NF-κB-MAPK pathway	Aggravated the inflammatory response Promoted the production of the proinflammatory factors TNF-α,IL-6, and IL-1β Reduced the survival rate of septic mice promoted LPS-induced macrophage polarization into a proinflammatory phenotypepromoted LPS-induced macrophage polarization into a proinflammatory phenotype.	(Gao et al., 2022)
50	Escherichia coli	ND	human microvascular endothelial cells	TLR4/NF-κB pathway	Induced the release of IL-8 Stimulated pulmonary inflammatory response with infiltration of neutrophils into the lungs	(Lee et al., 2018)
51	Escherichia (E.) coli	ND	human umbilical endothelial cells	NF-κB pathway	Increased expression of E-selectin and intercellular adhesion molecule Elevated Interleukin-6 levels	(Soult et al., 2013)
52	enterotoxigenic E. coli	ND	human umbilical vein endothelial cells	ND	Increased the expression of TF, E-selectin, and P-selectin Decreased the expression of thrombomodulin	(Soult et al., 2014)

ND, not described; eCIRP, extracellular cold-inducible RNA-binding protein; HMGB1, high-mobility group protein 1; LPS, Lipopolysaccharide; TNF-α, tumor necrosis factor; IL-1β, Interleukin-1 beta; IL-6, Interleukin-6; PMNs, Polymorphonuclear neutrophils; BMMSCs, bone marrow-derived mesenchymal stem cells; EPCs, endothelial progenitor cells; HMVECs, human microvascular endothelial cells; ADSCs, Adipose Tissue-Derived Stem Cells; HPMVEC, human pulmonary microvascular endothelial cell; MCP-1, monocyte chemoattractant protein-1; MMP-9, matrix metalloproteinase-9; RBCs, red blood cells.

### 3.1 EVs derived from host cells

#### 3.1.1 Immune cells

In sepsis, the immune system was activated, a variety of immune cells and immune molecules interact and form a complex regulatory network, and EVs are an important pathway for immune cell functional crosstalk. Here, we summarized the research progress of immune cell-derived EVs in sepsis.

##### 3.1.1.1 Neutrophils

Neutrophils are an important part of the innate immune system and act as first responders by migrating to the origin of inflammation. The concentration of neutrophil-derived extracellular vesicles was elevated in sepsis, both in the circulation and in inflammatory lesions (Prakash et al., 2012; Timár et al., 2013; Johnson et al., 2017; Chen et al., 2020). Neutrophil-derived extracellular vesicles in inflammatory lesions can modulate immune responses by activating phagocytes (Prakash et al., 2012). The concentration of neutrophil-derived extracellular vesicles in circulation was correlated with disease severity and IL-6, and have a certain value in the prognostic assessment of sepsis (Chen et al., 2020).

According to the production mechanism, neutrophil-derived EVs can be divided into two subtypes: neutrophil-derived microvesicles (NDMV) and neutrophil-derived trails (NDTR) (Hyun et al., 2012; Lim et al., 2015; Youn et al., 2021). Unlike NDMVs, which are thought to be produced by neutrophils in the inflammatory lesions, NDTRs are produced by the migration of neutrophils to the inflammatory lesions (Hyun et al., 2012; Lim et al., 2015; Youn et al., 2021). Furthermore, in contrast to the anti-inflammatory effect of NDMV, NDTR carried more pro-inflammatory miRNAs (eg. miR-1260, miR-1285, miR-4454, and miR-7975) and induced M1 polarization in macrophages (Youn et al., 2021). NDTR-treated CLP-septic mice showed increased survival, while NDMV did not (Youn et al., 2021). Recently, researchers have discovered a neutrophil-derived structure that is different from exosomes, and its formation may be related to the rolling of neutrophils on the vessel wall, similar to NDTRs discovered by previous investigators (Marki et al., 2021).

Most of the current studies have demonstrated that neutrophil-derived EVs have anti-inflammatory effects (Gasser and Schifferli, 2004; Eken et al., 2008; Dalli et al., 2008; Eken et al., 2010), and play a host protective role in sepsis, which is related to cargoes. In the blood of patients with sepsis, the expression levels of A2MG and CERU proteins were up-regulated (Dalli et al., 2013). A2MG-enriched extracellular vesicles enhanced neutrophil reactivity and promoted neutrophil adhesion to vascular endothelial cells, enhanced bacterial clearance, reduced inflammatory responses, and improved survival in septic mice (Dalli et al., 2014). EVs derived from neutrophils co-incubated with Staphylococcus aureus are enriched with a variety of antibacterial proteins and bind to bacteria to form large aggregates to isolate and immobilize bacteria. These two properties are beneficial to limit the growth of microorganisms in the early stage of infection and have an early antibacterial effect (Timár et al., 2013). Contrary to this, however, NDMP increased the intraperitoneal bacterial load in CLP mice, and decreased neutrophil recruitment, inhibited macrophage activation, thereby aggravated immunosuppression, and increased mortality in sepsis (Johnson et al., 2017).

##### 3.1.1.2 Monocytes and macrophages

In sepsis, monocyte- and macrophage-derived EVs are involved in pathological processes such as inflammation, immune regulation, organ damage, and coagulation through multiple pathways.

Studies have shown that in sepsis, monocyte/macrophage-derived EVs carried abundant specific cargoes that cause inflammatory responses and organ damage (Wang et al., 2019; Wisler et al., 2020; Sui et al., 2021b), and were involved in promoting disease progression. LPS-stimulated monocyte/macrophage-derived EVs carry a variety of DAMPs, such as HMGB1, histones, eCIRP, mtDAMPs etc. These DAMPs can promote inflammatory responses and neutrophil migration, trigger hepatocyte pyroptosis, and induce endothelial dysfunction (Nair et al., 2018; Wang et al., 2021; Murao et al., 2021; Konecna et al., 2021; Yang et al., 2022). The level of Gasdermin-D was increased in monocyte-derived extracellular vesicles (Homsy et al., 2019), and mediated apoptosis of human pulmonary microvascular endothelial cells through caspase 1, resulting in damage to the alveolar-capillary barrier (Mitra et al., 2015; Mitra et al., 2018). The expression of the TXNIP-NLRP3 complex in monocyte-derived exosomes caused sepsis-related cardiovascular inflammation and myocardial dysfunction by promoting the activation of IL-1β and Interleukin-18 (IL-18) in macrophages (Wang et al., 2021). Additionally, monocyte-derived tissue factor (TF)^+^MPs promoted coagulation by activating both intrinsic and extrinsic pathways (Woei et al., 2012; Oehmcke et al., 2012; Franks et al., 2013). Furthermore, the chemokine CXCL2-containing EVs released by macrophages recruited neutrophils and activated their CXCR2/PKC/NOX4 pathway *in vivo* and *in vitro*, promoting tissue damage (Wang et al., 2021). In addition to deleterious effects, macrophage-derived EVs were also protective against sepsis. Prdx4 was encapsulated into EVs released by activated macrophages, inhibited caspase-1 cleavage and IL-1β maturation, and attenuated cytokine release and inflammasome activation in sepsis (Lipinski et al., 2019). The P2X7 receptor of macrophages induced CD14 release from EVs, reduced CD14-dependent pro-inflammatory signaling in macrophages and bacterial dissemination, and improved survival during sepsis (Alarcón-Vila et al., 2020). Furthermore, TREM2 expressed on macrophages can inhibit the process of steroidogenesis in adrenal cortical cells mediated by macrophage-derived exosomes and improve tissue perfusion in septic shock (Ye et al., 2021).

##### 3.1.1.3 Dendritic Cells (DCs)

DCs are a class of immune cells with antigen-presenting properties that act as a bridge between the innate immune system and the adaptive immune system. Few studies have investigated the role of dendritic cell-derived EVs in sepsis. It was found that immature dendritic cells and mature dendritic cell-derived exosomes can provide essential milk fat globule-containing EGF factor VIII (MFGE8) for complete phagocytosis of apoptotic cells, reduced proinflammatory cytokine release, improved systemic inflammatory response in sepsis and decreased mortality (Miksa et al., 2006; Miksa et al., 2009).

#### 3.1.2 Non-immune cells

##### 3.1.2.1 Endothelial cells (ECs)

ECs are a highly dynamic cell layer that maintains homeostasis in physiological states. During infection, however, pathogen-associated molecular patterns (PAMPs) and DAMPs activated ECs and impaired their structure and functions (Ait-Oufella et al., 2010). In sepsis, ECs have pro-apoptotic, pro-inflammatory, pro-adhesive and pro-coagulant effects (Joffre et al., 2020).

In sepsis, the number of endothelial cell-derived EVs is increased (Wang et al., 2015; Chatterjee et al., 2020). They are involved in the regulation of inflammatory responses, endothelial barrier function, and antigen presentation. EVs released from ECs caused endothelial barrier dysfunction by impairing the integrity of endothelial cell adhesion junctions and cytoskeletal homeostasis and promoted endothelial inflammatory injury by promoting neutrophil-endothelial cell adhesion and neutrophil extracellular traps (Liu et al., 2017; Chatterjee et al., 2020). In contrast, Tu et al. found the anti-inflammatory effect of endothelial cell-derived exosomes, mainly dependent on HSPA12B, which inhibited the inflammatory response of macrophages by downregulating NF-κB activation and nuclear translocation (Tu et al., 2020). Human brain microvascular endothelial cell derived extracellular vesicles expressed molecules related to T cell stimulation and activation, including CD40, ICOSL, and MHC II, promoted T cell activation and proliferation, and activated adaptive immune responses (Wheway et al., 2014). Furthermore, endothelial cell-derived EVs protected multiple organ functions in sepsis, such as the lungs (Wang et al., 2015; Jiang et al., 2021) and heart (Cao et al., 2021). Its protective effect on the lung is dependent on the miR-125b-5p carried in EVs (Jiang et al., 2021).

Endothelial cell-derived EVs were also associated with activation of coagulation. In the early stage of septic shock, EMPs in the circulation of patients with DIC was increased, and this change occurred before activation of coagulation (Delabranche et al., 2013; Delabranche et al., 2016). ECs may participate in the sepsis-related coagulation process through TF^+^EVs (Del Turco et al., 2007; Matsumoto et al., 2015).

##### 3.1.2.2 Platelets

Platelet reactivity increased early in sepsis (Akinosoglou et al., 2017), but was depleted as the disease progresses (Claushuis et al., 2016; Thiery-Antier et al., 2016; Akinosoglou et al., 2017). Platelets promoted excessive inflammation, disseminated intravascular coagulation, and microthrombosis, which subsequently lead to multiorgan failure (de Stoppelaar et al., 2014). In sepsis, the number of platelet-derived extracellular vesicles increased (Woth et al., 2012; Tőkés-Füzesi et al., 2013; Wang et al., 2017; Wang et al., 2018), and it helps to assess the severity of septic shock and the occurrence of DIC, which is associated with septic shock mortality (Lehner et al., 2016; Boscolo et al., 2019). Studies have showed that platelet-derived extracellular vesicles are involved in disease progression (Barry et al., 1999; Brown and McIntyre, 2011; Sung et al., 2019).

Platelet-derived EVs bind to circulating immune cells (Fendl et al., 2018), not only transmited signaling molecules, but also regulated the functions of various immune cells. Dengue virus induced platelet-derived extracellular vesicles promotes pro-inflammatory cytokine release by activating TLR2 on macrophages (Sung et al., 2019). Staphylococcus superantigen-like protein 5 (SSL5) expressed by Staphylococcus aureus can induce the production of platelet-derived extracellular vesicles in bacterial infectious diseases. SSL5-platelet-derived extracellular vesicles mediate CD40/TRAF6/NFκB signaling pathway activation and stimulate monocytes to release inflammatory mediators (Bei et al., 2016). platelet-derived extracellular vesicles also inhibited the production of IL-17 by regulatory T cells *via* P-selectin (Dinkla et al., 2016). It was found that platelet-derived exosomes are also involved in promoting excessive NET formation in sepsis and subsequent organ damage (McDonald et al., 2014; Jiao et al., 2020). In addition to pro-inflammatory effects, platelet-derived extracellular vesicles also display strong procoagulant properties in sepsis, mainly by inducing thrombin formation through PS exposure and the intrinsic and extrinsic pathways of coagulation (Wang et al., 2018; Boscolo et al., 2019).

In sepsis, platelet-derived EVs were involved in the regulation of vascular endothelial function and multiple organ functions (Nomura et al., 2000; Janiszewski et al., 2004; Azevedo et al., 2007; Gambim et al., 2007). NADPH oxidase activity of platelet-derived exosomes in sepsis can induce caspase-3 activation and apoptosis of ECs by producing superoxide, NO and peroxynitrite, causing vascular dysfunction and cardiac dysfunction (Janiszewski et al., 2004; Gambim et al., 2007; Monteiro et al., 2017). In addition, the number of platelet-derived extracellular vesicles was negatively correlated with blood urea nitrogen and creatinine concentrations, which may be involved in sepsis-related renal impairment (Tőkés-Füzesi et al., 2013).

### 3.2 Pathogen-derived outer membrane vesicles (OMVs)

OMVs are nanoscale EVs shed from bacterial envelope (Horstman and Kuehn, 2000). As an effective mechanism for direct communication between bacteria and host cells, OMVs were involved in inducing host pathological changes and pathogens evading host immunity. OMVs can also be used as inanimate vaccine platforms to protect the host. We summarized the recent research progress of OMVs in the occurrence and development of sepsis.

Studies have found that OMVs usually carry virulence molecules from donor bacteria, such as ClyA protein (Wai et al., 2003), active enterotoxin (Kesty et al., 2004), and LPS (Vanaja et al., 2016; Gu et al., 2019), etc. These virulence molecules were delivered to host cells and cause inflammatory responses in the body (Yoon et al., 2011), possibly dependent on TLR2 or TLR4 (Park et al., 2010; Shah et al., 2012; Park et al., 2018). OMVs also transported LPS into the cytosol of host cells and subsequently activated caspase-11, triggering pyroptosis and caspase-1-activated cytosolic LPS sensing pathways (Vanaja et al., 2016; Gu et al., 2019). In addition, virulence molecules can be further assembled and enriched in OMVs, showing stronger virulence than the donor bacteria (Wai et al., 2003), and have strong pathogenicity to the body (Ellis and Kuehn, 2010; Kulp and Kuehn, 2010).

The transmission of OMVs is a way for pathogens to evade host immunity. Bap1 carried on the membrane of OMVs binds to antimicrobial peptides, reducing the concentration of free antimicrobial peptides, resulting in apparent resistance and survival of Vibrio cholerae (Duperthuy et al., 2013). In addition, OMVs containing OmpU can also bind to C1q *via* IgG, resulting in the inactivation of complement-mediated serum killing of the bacteria (Aung et al., 2016). Thus improved the survival of highly serum sensitive V. cholerae (Aung et al., 2016).

OMVs activated innate and adaptive immune responses in sepsis through multiple pathways. Alaniz et al. found that OMVs from Salmonella typhimurium potently activated macrophages and dendritic cells, increased the expression of MHC-II, CD86 and the production of proinflammatory mediators (Alaniz et al., 2007). E. coli OMVs recruited neutrophils to the lung by inducing IL-8/CXCL1 released from ECs (Lee et al., 2018). In addition to the innate immune response, OMVs induced activation of B cells and CD4(+) T cells by carrying specific Ags, leading to activation of adaptive immune (Alaniz et al., 2007; Vidakovics et al., 2010).. In addition, OMVs also initiated inflammatory cascade in ECs through the NF-κB pathway (Soult et al., 2013).

Studies have found that OMVs contributed to the hypercoagulable response in sepsis, leading to sepsis-related DIC (Wang et al., 2019). OMVs released by N. meningitidis increased the expression of TF and plasminogen activator inhibitor 2 on monocytes, which favors fibrin deposition in the monocyte microenvironment and causes DIC and microthrombosis (Mirlashari et al., 2001). OMVs activated ECs and promoted platelet activation during infection (Soult et al., 2014), and also induced DIC through the caspase-11-GSDMD pathway (Wang et al., 2019; Peng et al., 2020). In addition, OMV was involved in the development of sepsis-related cardiac dysfunction (Svennerholm et al., 2017).

Numerous studies have shown that OMV can be used as a vaccine to induce protective immunity against pathogenic bacterial infection (Unal et al., 2011; van der Pol et al., 2015; Yu et al., 2018). Kim et al. found that pre-exposure to sublethal doses of OMV was protective against sepsis. OMV-Ags promoted the production of IFN-γ and IL-17 of T cells, which can enhance bacterial clearance and inhibit OMV-induced systemic inflammation to prevent E. coli-induced lethality (Kim et al., 2013). Pretreatment of mice with multidrug-resistant Acinetobacter baumannii OMVs protect septic mice from challenge with homologous bacteria by both active and passive immunization (Huang et al., 2014). The application of genetic engineering technology enables OMVs to display complete heterologous proteins and induce specific antibody responses, which can be used as a vaccine platform against sepsis (Huang et al., 2016; Gerritzen et al., 2017). In the study of Huang et al., recombination of outer membrane protein Omp22 of Acinetobacter baumannii and E. coli-derived OMVs (Omp22-OMV) induced high titers of specific antibodies *in vivo*, and protected septic mice from lethal challenge with Acinetobacter baumannii strains (Huang et al., 2016). A study by Nieves et al. demonstrated that B. pseudomallei OMVs derived from strain 1026b were significantly protective against septic infection with B. pseudomallei strain K96243 (Nieves et al., 2014).

### 3.3 Mesenchymal stem cells (MSCs)/EPCs

MSCs are a class of adult stem cells that are widely present in various human tissues and have the potential for multi-directional differentiation and play a protective role in sepsis (Walter et al., 2014). It has been found that MSC-derived EVs (MSC-EVs) can act as a way for MSCs to secrete signaling molecules, exerting similar therapeutic benefits as MSCs (Rani et al., 2015).

#### 3.3.1 Bone marrow mesenchymal stem cells (BMMSCs)

Current studies have shown that BMMSC-EVs can effectively improve sepsis-related inflammatory response, protect organ function, and improve survival. BMMSC-EVs were able to promote macrophage anti-inflammatory effects and attenuate systemic inflammatory responses in sepsis (Park et al., 2019), which may be related to the miR-27b, miR-223 and miR-191 they carried (Wang et al., 2015; Sun et al., 2021; Liu et al., 2022). In addition, lncRNA-p21 and lncRNA IGF2-AS in BMMSC-EVs are involved in alleviating sepsis-related lung injury and promoting endothelial progenitor cell pyroptosis, respectively (Sui et al., 2021a; Liang et al., 2022).

The study by Jae et al. showed that keratinocyte growth factor carried by human BMMSC-EVs could alleviate lung inflammation and pathological damage, alleviate pulmonary edema, reduce bacterial load, and improve the survival rate of E. coli pneumonia ALI mice (Zhu et al., 2014; Monsel et al., 2015). Subsequent experiments showed that MSC-EVs exerted the same lung protection in an *in vitro* perfused human lung model of bacterial pneumonia (Park et al., 2019). *In vitro*, MSC-EVs enhanced the phagocytosis of bacteria by human monocytes, inhibited the secretion of inflammatory factors, and restored ATP levels in damaged alveolar epithelial type 2 cells (Monsel et al., 2015). The current mechanistic study found that the functional regulation of monocyte-macrophages by BMMSC-EVs depends on multiple pathways. Such as the regulatory effect of Ang-1 (Tang et al., 2017), enhancing macrophage oxidative phosphorylation through functional mitochondria (Morrison et al., 2017), inhibiting hypoxia-inducible factor 1α-dependent cellular glycolysis process (Deng et al., 2020), regulating Nrf -2/ARE and nuclear factor -κB (NF-κB) axis signal transduction, etc. (Li et al., 2020).

#### 3.3.2 Umbilical cord mesenchymal stem cells (UCMSCs)

Human umbilical cord MSC-derived exosomes (hucMSC-Ex) inhibited NF-κB activity *via* the miR-146b/IRAK1 axis, attenuated sepsis-related acute kidney injury and improved survival in mice with sepsis (Zhang et al., 2020). *In vitro* experiments showed that hucMSC-Ex could inhibit LPS-induced macrophage M1 polarization and promote M2 polarization (Song et al., 2019), and this anti-inflammatory effect may depend on the miR-181c/TLR regulatory axis (Li et al., 2016). Exosomes derived from MSCs pretreated with the proinflammatory cytokine Interleukin-1 beta (IL-1β) showed stronger anti-inflammatory effects (Song et al., 2017). Another study showed that hucMSC-Ex may improve sepsis-related acute lung injury by inhibiting the phosphorylation of mitogen-activated protein kinase (Yang et al., 2017).

#### 3.3.3 Adipose-derived stem cellsand dental pulp stem cells (DPSC)

ADSCs-derived EVs can attenuate systemic inflammatory response, organ damage, and improve survival in sepsis (Chang et al., 2018; Chang et al., 2019). Its anti-inflammatory effect mainly depends on Nrf2/HO-1 axis, SIRT1/NF-κB signaling pathway, Notch-miR148a-3p axis, etc (Shen et al., 2021; Gao et al., 2020; Bai et al., 2020). In addition, it also exerted a protective effect on endothelial cells (ECs) through the miR-126-PI3K/Akt pathway and alleviated histone-induced acute lung injury (Mizuta et al., 2020). mmu_circ_0001295 in exosomes of ADSCs pretreated with hypoxia was involved in alleviating sepsis-related renal injury (Cao et al., 2022).

Human DPSCs-derived exosomes have a protective effect on LPS-induced acute lung injury, and the mechanism may be related to the inhibition of MAPK and the activation of the NF-κB signaling pathway (Su et al., 2018).

#### 3.3.4 Endothelial progenitor cells (EPCs)

EPCs are precursor cells of vascular endothelial cells that maintain vascular homeostasis and promote vascular repair in pathological conditions (Urbich and Dimmeler, 2004). Multiple studies have found that EPC-derived EVs are protective against sepsis (Zhou et al., 2018; Zhou et al., 2019; He et al., 2020; Ma et al., 2021). It has been demonstrated that EPC-derived exosomal miR-126 mediates the prevention of sepsis microvascular dysfunction and improves lung and kidney function (Zhou et al., 2018). Among the lung-protective effects, miR-126-3p and miR-126-5p increased the expression of epithelial tight junction protein, while reduced ALI-related target genes, thereby maintained the integrity of the alveolar epithelial barrier and reduced lung inflammation and tissue damage (Zhou et al., 2019). Its renoprotective effect was dependent on the regulation of the KDM6B/H3K27me3/TNF-α axis by miR-93-5p (He et al., 2020). Furthermore, EPC-derived EVs promote macrophage M2 polarization to alleviate sepsis by delivering the lncRNA TUG1 (Ma et al., 2021).

EVs from MSCs and EPCs play a protective role in sepsis through different pathways, and the exploration of their regulatory mechanisms will help provide a molecular biological basis for cell-free therapy and precision therapy of sepsis.

## 4 Role of EVs in the pathogenesis of sepsis

### 4.1 Immune regulation

The host’s immune response to pathogens begins with the recognition of pathogens. PAMPs from bacteria were assembled and enriched in OMVs (Wai et al., 2003; Kesty et al., 2004; Vanaja et al., 2016; Gu et al., 2019), and recognized by pattern recognition receptors (PRRs) on the host cell surface or in the cytoplasm (Park et al., 2010; Shah et al., 2012; Park et al., 2018). Then triggered activation of intracellular signaling pathways and activates key transcription factors such as NF-κB and activator protein 1 (AP-1) and interferon regulatory factor (IRF), which regulate inflammation reaction (Takeuchi and Akira, 2010). In addition, PAMPs of OMVs entering the cytosol also activated caspase 1 and caspase 11, which promoted inflammasome activation, mediated the maturation and release of IL-1β and IL-18, and triggered pyroptosis (Lamkanfi and Dixit, 2014; Broz et al., 2020; Dhital et al., 2021).

In sepsis, activated cells produced EVs carrying DAMPs, such as HMGB1 (Li et al., 2020; Jiao et al., 2020), histones (Nair et al., 2018), and ATP (Sakaki et al., 2013). EVs released into the circulation delivered DAMPs to distant host cells, triggering inflammatory cascades (Nair et al., 2018; Murao et al., 2021), cell death (Li et al., 2020; Wang et al., 2021), increased endothelial permeability (Yang et al., 2022), and NET formation (Jiao et al., 2020). EVs induced pro-inflammatory responses by activating different PRRs and different signaling pathways, such as inducing the secretion of pro-inflammatory factors, promoting macrophage proliferation and M1 polarization. The regulatory effect mainly depend on the cargo of EVs (Sakaki et al., 2013; Xu et al., 2018; Park et al., 2018; Jiang et al., 2019; Lv et al., 2020). In addition to activating the innate immune response, EVs in sepsis also induced the differentiation of Th1/Th2 cells and enhanced T lymphocyte proliferation and migration, activating the adaptive immune response (Gao et al., 2019).

Transfusion of red blood cell suspensions is an important treatment for critically ill patients, but it has been found that it may aggravate the inflammatory response in sepsis (Wu et al., 2017). This pro-inflammatory response may be associated with EVs from stored erythrocytes (Straat et al., 2016; Gao et al., 2022). Erythrocyte-derived EVs may amplify inflammation through thrombin-dependent complement activation (Zecher et al., 2014; Almizraq et al., 2016; Fischer et al., 2017). *In vitro*, erythrocyte-derived EVs induced the M1 polarization of macrophages and increased the release of pro-inflammatory cytokines, the underlying mechanism may be EVs-mediated upregulation of TLR4-MyD88-NF-κB-MAPK activity (Gao et al., 2022).

In sepsis, inflammatory responses co-occur with immunosuppression, inflammatory responses were associated with multiple organ failure and early death, whereas anti-inflammatory responses were associated with reactivation of underlying viral infection and delayed death (van der Poll et al., 2021). Immunosuppression was characterized by impaired function of multiple immune cells and reduced production of proinflammatory cytokines (van der Poll et al., 2021). Studies have found that external EVs derived from MSCs were also involved in the process of immunosuppression in sepsis, induced M2 polarization, inhibited M1 polarization, and reduced the production of inflammatory factors through miR-146a, miR-21, Nrf2/HO-1 axis, and Notch-miR148a-3p axis, respectively (Song et al., 2017; Bai et al., 2020; Shen et al., 2021; Yao et al., 2021). Furthermore, endothelial exosomal HSPA12B inhibited NF-κB activation and suppressed the inflammatory response of macrophages (Tu et al., 2020). However, studies have also shown that EVs were involved in relieving T cell-related immunosuppression. In sepsis, exosomes ameliorated LPS-induced apoptosis of T lymphocytes by inhibiting Bad *via* hsa-miR-7-5p (Deng et al., 2019).

The imbalance of inflammatory response and immunosuppression plays a pivotal role in the occurrence and development of sepsis. EVs may modulate the balance between pro-inflammatory responses and immunosuppression. However, there were few studies on the role of EVs in the immunosuppressive mechanism of sepsis. Revealing their signal transduction mechanism will help deepen the understanding of the immune regulation of sepsis and provide ideas for the immunotherapy of sepsis.

### 4.2 Endothelial dysfunction

ECs regulate vascular barrier function, coagulation pathways, leukocyte adhesion, and vasomotor tone in physiological conditions (Joffre and Hellman, 2021). However, ECs were modified to pro-apoptotic, pro-inflammatory, pro-adhesive, and pro-coagulant phenotypes in sepsis (Joffre et al., 2020).

In sepsis, EVs released by various activated cells can lead to ECs damage or apoptosis. EVs derived from EPCs may regulate endothelial barrier integrity through miRNAs they carry (Goodwin et al., 2015). EVs released by activated ECs can modulate the barrier function of themselves through contractile cytoskeleton reorganization and dissociation of adherent junctions (Chatterjee et al., 2020). In addition, EVs derived from activated neutrophils, monocytes and platelets induced endothelial cell injury and apoptosis *via* the myeloperoxidase-hydrogen peroxide-chloride system, GSDMD/caspase-1 and active ROS/RNS, respectively (Janiszewski et al., 2004; Gambim et al., 2007; Pitanga et al., 2014; Mitra et al., 2018). Circulating EVs from pathogens or host cells can also activate the inflammatory pathway of endothelial cells and increase their TF expression, showing pro-inflammatory and procoagulant abilities (Soult et al., 2014; Yang et al., 2022). Endothelial dysfunction caused by EVs in sepsis may impair microcirculatory blood flow, reduce tissue perfusion and even lead to impaired organ function (Joffre et al., 2020).

### 4.3 Coagulation disorders

The occurrence of DIC in sepsis significantly increased the mortality rate (Gando et al., 2019). Studies have shown that EVs derived from pathogens and host cells in sepsis aggravated coagulopathy or DIC (Park et al., 2010; Zafrani et al., 2012). E. coli OMV induced coagulation in a TLR4-dependent manner (Wang et al., 2019), and mediated activation of the coagulation cascade by increasing TF activity through the caspase-11-GSDMD pathway in sepsis (Peng et al., 2020). OMVs are also able to activate ECs, which have elevated TF expression and activated platelets, leading to hypercoagulability in sepsis (Soult et al., 2014). Endothelial extracellular vesicles were closely related to early DIC (Delabranche et al., 2013; Delabranche et al., 2016), and the platelet-derived extracellular vesicles/platelet ratio can be used to assess the incidence of DIC (Boscolo et al., 2019).

Current studies suggested that circulating EVs in sepsis patients promotes coagulation through multiple mechanisms. It was found that the production of both circulating PS+ extracellular vesicles and PS+ platelet-derived extracellular vesicles was increased in sepsis, and PS exposed on the surface of extracellular vesicles induced coagulation activity in sepsis by promoting the generation of thrombin (Oehmcke et al., 2012; Mooberry et al., 2016; Wang et al., 2018; Zhang et al., 2016; Vance and Steenbergen, 2005). Circulating TF is the primary initiator of the extrinsic coagulation pathway and plays a central role in the development of coagulation disorders during sepsis (Tang et al., 2021). Studies have shown that TFs expressed on the surface of both ECs and monocyte-derived EVs were increased in severe sepsis (Matsumoto et al., 2015; Oehmcke et al., 2012). After exposure to E. coli or LPS, the number of circulating TF+ extracellular vesicles were increased, and the activity of TF+ extracellular vesicles were correlated with disease severity and the thrombin-antithrombin complex (TAT) (Woei et al., 2014; Wang et al., 2009). In addition, Neisseria meningitidis (Nm) and methicillin-resistant staphylococcus aureus (MRSA) can also induce the production of TF+ extracellular vesicles (Stephens et al., 2007; Franks et al., 2013). Nm induced the expression of TF mainly depend on LPS and activating complements C5 and C5a (Stephens et al., 2007; Øvstebø et al., 2014). Nieuwland et al. reported that extracellular vesicles expressing CD14 and TF were detected in the plasma of patients with Nm sepsis with severe DIC (Nieuwland et al., 2000), and the procoagulant activity of TF+ extracellular vesicles were correlated with the level of LPS in plasma (Hellum et al., 2014). Extracellular vesicles in sepsis also enhanced thrombin production and shorten clotting time in an FXI-dependent manner (Mooberry et al., 2016).

### 4.4 Circulatory abnormalities

In sepsis, circulatory dysfunction occur with the progression of the disease, and its pathophysiological characteristics include decreased vascular reactivity, vasodilation, microcirculation dysfunction, and abnormal cellular oxygen metabolism caused by circulatory disorders (Singer et al., 2016). In septic shock, increased circulating EVs are associated with microvascular occlusion, possibly related to microthrombosis, endothelial injury, and decreased erythrocyte deformability (Boisramé-Helms et al., 2014; Subramani et al., 2018). A study by Mortaza et al. found that leukocyte-derived EVs inhibited endothelial nitric oxide synthase activation and enhanced inducible nitric oxide synthase (iNOS) expression *in vivo*, which caused NO overproduction, induced systemic vasodilation, and led to lower mean arterial pressure in septic shock (Mortaza et al., 2009).

However, several studies have shown that EVs play a protective role in vascular function in sepsis. This protective effect may prevent hypotension in septic shock by preventing decreased vascular reactivity through the production of thromboxane A2 (Mostefai et al., 2008). Another study found that its protective effect on blood vessels may be related to enhanced IL-10 expression (Mostefai et al., 2013). Extracellular vesicles of septic rats pretreated with activated protein C (aPC) have increased thromboxane content and aPC activity, inhibiting iNOS production, which is beneficial for improving hemodynamics (Boisramé-Helms et al., 2014). Therefore, the effective utilization or modification of EVs may be a potential therapeutic measure to correct circulatory dysfunction in sepsis.

### 4.5 Organ damage

Severe sepsis is often complicated by multiple organ system dysfunctions. Here, we discussed the role EVs play in sepsis-related organ dysfunction.

#### 4.5.1 Acute respiratory distress syndrome (ARDS)

ARDS is a common organ dysfunction in sepsis. In sepsis-related ARDS, the number of EVs in both the bronchoalveolar lavage fluid (BALF) and the circulation is increased (Letsiou et al., 2015; Li et al., 2015; Lee et al., 2018). The EVs in BALF are mainly derived from alveolar macrophages (Lee et al., 2018), and the circulating EVs are mainly from ECs and leukocytes (Letsiou et al., 2015; Li et al., 2015; Takei et al., 2019; Danilov et al., 2001; Takei et al., 2019).

In sepsis, EVs promoted the pathogenesis of sepsis-associated ARDS (Sui et al., 2021b). BALF-EVs promoted the recruitment of macrophages to the lung and release of inflammatory factors (Lee et al., 2018), increased epithelial cell inflammatory response, and reduced the expression of tight junction protein ZO-1, impairing the epithelial barrier (Yuan et al., 2018). Circulating EVs increased pulmonary macrophage M1 activation and induced ARDS-related pathological changes such as pulmonary neutrophil infiltration, alveolar hemorrhage, and early hyaline membrane formation (Jiang et al., 2019; Li et al., 2015). *In vitro*, EVs from activated macrophages can activate resting macrophages, mediate macrophage recruitment to the lung, and promote inflammatory responses (Lee et al., 2018; Li et al., 2018). When stimulated by LPS, EVs from ECs and monocytes damage ECs through sphingosine-1-phosphate receptor 3 (SIPR3) and caspase 1, respectively, resulting in endothelial barrier disruption (Sun et al., 2012; Mitra et al., 2015; Mitra et al., 2018). The inflammatory response mediated by EVs may be related to the signaling of the miR-145/TGFBR2 axis and the miR-210-30/ATG7 axis (Cao et al., 2019; Li et al., 2021). In addition, EVs also caused vascular endothelial barrier dysfunction through miR-1-3p/SERP1, causing lung injury (Gao et al., 2021).

Studies have also found that high levels of EVs are associated with better prognosis in ARDS, suggesting that EVs in sepsis may have a protective effect on ARDS (Soriano et al., 2005; Guervilly et al., 2011; Shaver et al., 2017). Mesenchymal stem cell-derived EVs interact with immune cells or stromal cells associated with acute lung injury, including inhibition of alveolar epithelial cell proliferation and inflammatory response (Li et al., 2020; Deng et al., 2022); inhibition of pulmonary vascular endothelial cell apoptosis, improvement of endothelial barrier (Chang et al., 2018; Mizuta et al., 2020); inhibition of alveolar macrophage M1 polarization and promotion of M2 polarization, reducing inflammation reaction (Deng et al., 2020). In addition, endothelial progenitor cell-derived EVs are also involved in improving the alveolar epithelial barrier and reducing inflammatory infiltration in the lungs (Zhou et al., 2019).

#### 4.5.2 Myocardial dysfunction

Sepsis-induced myocardial dysfunction (SIMD) is a fatal symptom in patients with sepsis (Liu et al., 2017). Studies have found that OMVs derived from pathogens not only reduced the viability of cardiomyocytes but also promoted the infiltration of inflammatory cells into the myocardium and induces the release of inflammatory cytokines from macrophages, resulting in cardiac damage and decreased cardiac function (Svennerholm et al., 2017). In addition, EVs from host cells also mediate myocardial dysfunction (Essandoh et al., 2015). Wang et al. found that monocyte-derived exosomes delivered the TXNIP-NLRP3 complex to heart-resident macrophages, where they activated caspase-1 and cleaved inactive IL-1β and IL-18 (Wang et al., 2021). EVs also impaired myocardial function and induced septic myocardial dysfunction through a redox-dependent pathway (Azevedo et al., 2007; Mu et al., 2018). There are also some studies showing that EVs delivered to cardiomyocytes attenuated inflammation and cardiomyocyte death *via* miR-223 and miR-126, respectively, and reduce sepsis-induced heart failure and mortality (Wang et al., 2015; Zhang et al., 2020).

#### 4.5.3 Acute kidney injury (AKI)

In sepsis, EVs can serve as diagnostic markers of acute kidney injury and help assess its severity. In detail, the expression levels of uATF protein in urinary exosomes may serve as a biomarker for septic AKI (Panich et al., 2017). Increased numbers of platelet-derived extracellular vesicles were negatively correlated with blood urea nitrogen and creatinine concentrations (Tőkés-Füzesi et al., 2013). Most of the current studies have shown that EVs were involved in the renal protection of sepsis through multiple pathways. EVs derived from MSCs may attenuate the inflammatory infiltration of kidneys through the SIRT1 signaling pathway and the miR-146b/IRAK1/NF-κB signaling pathway (Chang et al., 2018; Gao et al., 2020; Zhang et al., 2020). Endothelial progenitor cell-derived EVs delivered miR-93-5p to renal tubular epithelial cells and attenuated vascular leakage, inflammation, and apoptosis through the KDM6B/H3K27me3/TNF-α axis (He et al., 2020). The role of EVs in septic AKI was also related to the macrophage phenotype. The study by Juan et al. revealed that M1 macrophage-derived exosomes promoted renal epithelial cell pyroptosis, while M2 macrophage-derived exosomes carried miR-93-5p, which inhibited renal epithelial cell pyroptosis and alleviated AKI by regulating TXNIP (Juan et al., 2021).

#### 4.5.4 Central nervous system dysfunction

EVs play an important role in the communication between blood and cerebrospinal fluid as a novel way of blood-brain communication, and are involved in maintaining brain homeostasis during endogenous toxins attack (Balusu et al., 2016; Shulyatnikova and Shavrin, 2021). After exposure to LPS stimulation, CPEs secrete EVs containing inflammation-related proteins and miRNAs (miR-146a and miR-15), which transmit inflammatory messages to the brain parenchyma through the cerebrospinal fluid (Balusu et al., 2016). The expression of serum exosomal NEAT1 was upregulated in a CLP rat model, possibly promoting ferroptosis by regulating the miR-9-5p/TFRC and GOT1 axes, thereby exacerbating sepsis-associated encephalopathy (Wei et al., 2022). In addition, EVs also play a cerebral protective role in sepsis. Exosomes derived from ADSCs significantly protected inflammatory infiltration and organ damage in the brain of a CLP rat model (Chang et al., 2019).

#### 4.5.5 Other organ dysfunction

Acute liver injury and intestinal mucosal inflammation are also common complications during sepsis, but little is known about the role of EVs in their development. In sepsis, macrophage-derived EVs mediated acute liver injury by triggering hepatocyte pyroptosis through the NLRP3 inflammasome (Wang et al., 2019; Wang et al., 2021). Intestinal epithelial -derived EVs inhibited intestinal mucosal inflammation *in vivo* (Appiah et al., 2020).

## 5 Conclusion and further prospects

The regulatory roles of EVs in sepsis are complex and diverse. With the development and application of omics technology, more and more studies have found that the expression profile of cargo carried by EVs in sepsis is dynamic, which helps correlate protein expression, RNA expression, and metabolic alterations in EVs with specific clinical features. Approaches to stratifying patients with sepsis according to biochemical and/or immunological profiles are critical for personalizing treatment.

EVs of different cell origins play different roles in sepsis, which may be related to the function of the parental cells. The regulatory role of activated immune cell-derived EVs in sepsis is two-sided, not only promoting the inflammatory cascade leading to tissue damage, but also reducing sepsis-related inflammation and organ dysfunction; Endothelial and platelet-derived EVs play important roles in inflammation, coagulation cascade, and inflammation-coagulation crosstalk; Pathogen-derived OMV damages host cells through its own virulence on the one hand, and activates the host immune system to cause tissue damage on the other hand. It can also be used as a vaccine to protect the host by activating active immunity; MSC- and EPC-derived EVs mainly suppress inflammatory responses and alleviate sepsis-related organ damage.

The pathophysiological changes of sepsis are dynamic, and EVs released by multiple activated cells can synergistically lead to specific pathophysiological changes, such as endothelial dysfunction, coagulation abnormalities, circulatory dysfunction and organ dysfunction. EVs can not only aggravate sepsis-related pathological changes, but also exert protective effects through different mechanisms. Sepsis treatment strategies based on EVs can be developed in the following ways. a. For EVs that aggravated the pathophysiological process of sepsis, specific inhibitors can be developed for precise blocking. b. Engineered EVs can be used as vaccines to stimulate active immunity and protect the body. c. EVs can be used as drug delivery vehicles. d. Exogenous MSC or EPC-derived EVs can be used as an effective treatment for sepsis. EVs carrying antibiotics and other drugs chemotaxis to the lesions actively and mediate the stable release of drugs. In conclusion, the regulatory role of EVs in sepsis is closely related to the cargoes they carry and their cellular origin. Exploring its regulatory mechanism in sepsis can provide a theoretical basis for the diagnosis, treatment strategy and vaccine prevention of sepsis in the future.

## Author contributions

All authors listed have made a substantial, direct, and intellectual contribution to the work. All authors read and approved the final manuscript.

## Glossary

**Table:**

EVs	Extracellular vesicles
MPs	Microparticles
CRP	C-reactive protein
HMGB1	High mobility group box 1 protein
eCIRP	Extracellular cold-induced RNA-binding protein
CPE	Choroid plexus epithelium
MSCs	Mesenchymal stem cells
BMMSCs	Bone marrow mesenchymal stem cells
MSC-EVs	MSC-derived EVs
UCMSCs	Umbilical cord Mesenchymal Stem Cells
hucMSC-Ex	Human umbilical cord MSC-derived exosomes
ADSCs	Adipose-derived stem cells
DPSCs	Dental pulp stem cells
EPCs	Endothelial progenitor cells
ECs	Endothelial cells
NDMV	Neutrophil-derived microvesicles
NDTR	Neutrophil-derived trails
IL-1β	Interleukin-1 beta
IL-18	Interleukin-18
Mac-EVs	Macrophage-derived EVs
DCs	Dendritic Cells
MFGE8	Milk fat globule-containing EGF factor VIII
DAMPs	Damage associated molecular patterns
PAMPs	Pathogen-associated molecular patterns
TNF-α	Tumor necrosis factor
SSL5	Staphylococcus superantigen-like protein 5
OMVs	Outer membrane vesicles
Bap1	Biofilm-associated extracellular matrix protein
NFKB	Nuclear factor -Kb
AP-1	Activator protein 1
IRF	Interferon regulatory factor
PRRs	Pattern recognition receptors
TF	Tissue factor
PS	Phosphatidylserine
TAT	Thrombin-antithrombin complex
Nm	Neisseria meningitidis
MRSA	Methicillin-resistant Staphylococcus aureus
MMP-10	Matrix metalloproteinase-10
iNOS	Inducible nitric oxide synthase
aPC	Activated protein C
ARDS	Acute respiratory distress syndrome
AKI	Acute kidney injury
BALF	Bronchoalveolar lavage fluid
ACE	Angiotensin-converting enzyme
SIPR3	Sphingosine-1-phosphate receptor 3
SIMD	Sepsis-induced myocardial dysfunction
CSF	Cerebrospinal fluid
